# The role of cholesterol and sphingolipids in chemokine receptor function and HIV-1 envelope glycoprotein-mediated fusion

**DOI:** 10.1186/1743-422X-3-104

**Published:** 2006-12-22

**Authors:** Sherimay Ablan, Satinder S Rawat, Mathias Viard, Ji Ming Wang, Anu Puri, Robert Blumenthal

**Affiliations:** 1 Center for Cancer Research Nanobiology Program, Center for Cancer Research, National Cancer Insitute, National Institutes of Health, Frederick, Maryland, USA; 2 Laboratory of Molecular Immunoregulation, Center for Cancer Research, National Cancer Insitute, National Institutes of Health, Frederick, Maryland, USA

## Abstract

**Background:**

HIV-1 entry into cells is a multifaceted process involving target cell CD4 and the chemokine receptors, CXCR4 or CCR5. The lipid composition of the host cell plays a significant role in the HIV fusion process as it orchestrates the appropriate disposition of CD4 and co-receptors required for HIV-1 envelope glycoprotein (Env)-mediated fusion. The cell membrane is primarily composed of sphingolipids and cholesterol. The effects of lipid modulation on CD4 disposition in the membrane and their role in HIV-1 entry have extensively been studied. To focus on the role of lipid composition on chemokine receptor function, we have by-passed the CD4 requirement for HIV-1 Env-mediated fusion by using a CD4-independent strain of HIV-1 Env.

**Results:**

Cell fusion mediated by a CD4-independent strain of HIV-1 Env was monitored by observing dye transfer between Env-expressing cells and NIH3T3 cells bearing CXCR4 or CCR5 in the presence or absence of CD4. Chemokine receptor signaling was assessed by monitoring changes in intracellular [Ca^2+^] mobilization induced by CCR5 or CXCR4 ligand. To modulate target membrane cholesterol or sphingolipids we used Methyl-β-cyclodextrin (MβCD) or 1-phenyl-2-hexadecanoylamino-3-morpholino-1-propanol (PPMP), respectively. Treatment of the target cells with these agents did not change the levels of CD4 or CXCR4, but reduced levels of CCR5 on the cell surface. Chemokine receptor signalling was inhibited by cholesterol removal but not by treatment with PPMP. HIV-1 Env mediated fusion was inhibited by >50% by cholesterol removal. Overall, PPMP treatment appeared to slow down the rates of CD4-independent HIV-1 Env-mediated Fusion. However, in the case of CXCR4-dependent fusion, the differences between untreated and PPMP-treated cells did not appear to be significant.

**Conclusion:**

Although modulation of cholesterol and sphingolipids has similar effects on CD4 -dependent HIV-1 Env-mediated fusion, sphingolipid modulation had little effect on CD4-independent HIV-1 Env-mediated fusion. Chemokine receptor function remained intact following treatment of cells with PPMP. Therefore such treatment may be considered a more suitable agent to inhibit CD4 dependent HIV-1 infection.

## Background

HIV-1 delivers its genetic material into the cell by direct fusion of the viral membrane with the plasma membrane of the host cells [1]. HIV-1 Env encodes a polypeptide (gp160) that is folded in the endoplasmic reticulum into a complex, disulphide-linked structure, which is anchored in the membrane by virtue of the hydrophobic, membrane-spanning domain in the gp41 moiety[2]. During post-translational processing, the precursor glycoproteins oligomerize and are extensively glycosylated before cellular proteinases cleave the precursor at a characteristic sequence to create the mature glycoproteins gp120 and gp41[3]. The surface glycoprotein, gp120, forms trimeric spikes[4], which are associated by non-covalent interactions with each subunit of the membrane-anchored gp41[5].

The triggering mechanisms that activate Env are quite complex involving target cell CD4 [6] and chemokine receptors CCR5 and CXCR4 [7]. The current data support a two-step model for receptor engagement that involves initial interactions between gp120 and CD4, followed by conformational changes in Env, which permit interaction of the gp120-CD4 complex with co-receptor leading to a further barrage of conformational changes that eventually lead to gp41 6-helix bundle formation and fusion [1]. Receptors in membranes are not randomly distributed and lipids can play an important role in choreography of receptors [8]. Therefore it stands to reason that modulation of lipid composition may affect the HIV-1 Env-mediated fusion reaction by altering the two-step choreography of CD4 and chemokine receptors required for HIV-1 entry. Based on this hypothesis a plethora of publications have appeared indicating that lipid modulation may have an indirect effect on HIV-1 entry/fusion by interfering with the choreography of CD4 and co-receptors (for reviews see [9,10].

In order to further examine the effect of lipids on HIV-1 entry we asked the question whether similar lipid modulation would affect HIV-1 entry/fusion when we consider just one receptor required for entry. We addressed these issues by performing studies with CD4-independent HIV-1 Envs whose chemokine receptor-binding site is exposed without prior interaction with CD4. Hoxie and coworkers derived a variant of HIV-1/IIIB, termed 8x, which acquired the ability to utilize CXCR4 without CD4 [11]. Moreover, when the V3 loop of a CCR5-tropic Env was substituted for its counterpart in the 8x Env, the resulting chimera (8xV3BAL) was found to utilize CCR5 but remained CD4 independent [11]. We have used these constructs to further explore the role of target membrane cholesterol and glycosphingolipids in HIV-1 Env-mediated fusion.

## Results

### Removal of cholesterol

**Figure 1:**
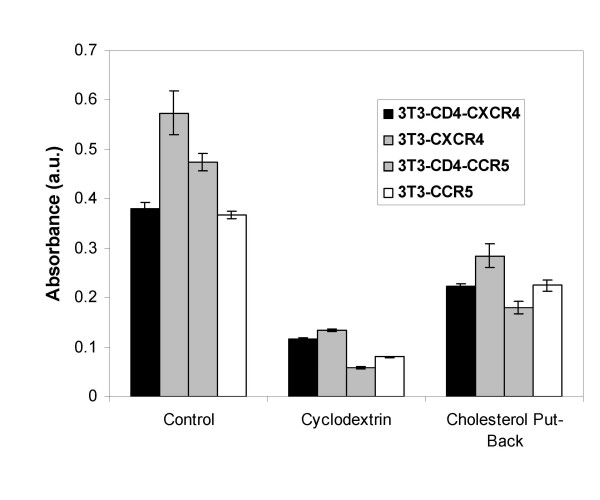
**Cholesterol Levels in NIH3T3 cells after MβCD treatment**. NIH 3T3 cells were treated with 10 mM MβCD for 30 minutes at 37°C and cholesterol in total cell lysate was determined as described previously [13]. For the put-back experiments the cholesterol-depleted cells were incubated with an MβCD-cholesterol mixture for 60 minutes at 37°C according to [13].

**Figure 2:**
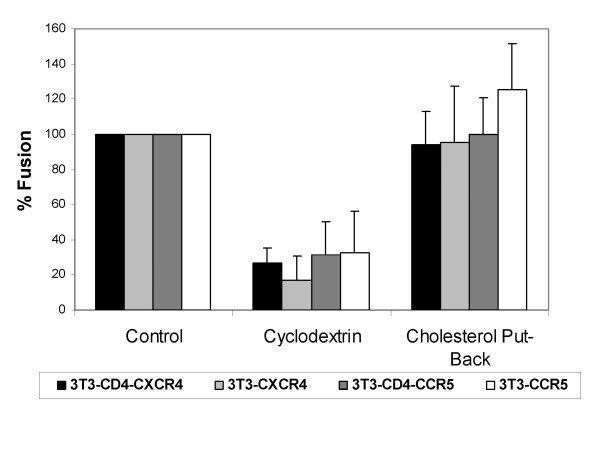
**The effect of cholesterol depletion from NIH3T3 Targets on CD4-independent HIV-1 Env-mediated fusion**. CMFDA-labeled cells (2 × 10^5 ^per well) expressing HIV-1_8x _(black and light gray bars) or HIV-1_8xV3BAL _(dark gray and white bars) Envs were added to the CMTMR-labeled targets expressing cognate co-receptors. The samples were incubated for 6 hrs at 37°C before, after and following cholesterol put-back as described in the legends to Figure 1. Fusion was determined as described in materials and methods.

To remove cholesterol from cells, we used the cholesterol-solubilizing agent methyl-β-cyclodextrin (MβCD), which stimulates cholesterol efflux from cells [12]. Figure 1 shows that incubation of NIH3T3 cells with 10 mM MβCD for 30 min at 37°C reduced cholesterol levels of the cells to about 22% of untreated controls. There was no significant effect on cell viability (not shown) or surface expression of CD4 and coreceptors [13]. Figure 2 shows that fusion mediated by CD4-independent HIV-1 Env was reduced to about 30% of untreated controls for both 8x and 8xV3BAL Envs, irrespective of the presence of CD4 in the target membrane. When these cells were replenished with cholesterol by incubation with pre-formed cholesterol- MβCD complexes, HIV-1 env-mediated fusion was fully recovered. Interestingly, in spite of the fact that cholesterol levels were only replenished to 50% of untreated control (Figure 1), HIV-1 Env-mediated fusion was fully recovered (Figure 2). This observation is consistent with the notion that the dependence of membrane organization on cholesterol is not linear, but rather that there is a critical concentration of cholesterol below which phase separation of domains occurs [14].

### Modulation of sphingolipid metabolism

**Figure 3:**
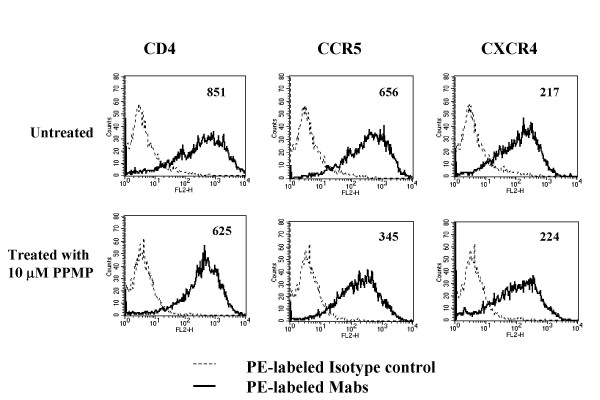
**Effect of PPMP Treatment on CD4 and Co-receptor Expression in NIH3T3 Cells**. NIH3T3 target cells on 12 well plates (5 × 10^4 ^per well) were cultured in the presence of 10 μM PPMP for 48–72 hours. CD4, CXCR4 and CCR5 levels before and after treatment with PPMP were determined by FACS using PE-conjugated Mabs as described in materials and methods. The numbers in the boxes respresent means of the fluorescence histograms.

To modulate shingolipid metabolism, we used an inhibitor of GlcCer synthase, PPMP [15]. The effects of such treatment was monitored by observing changes in the expression of cell surface glycosphingolipids by flow cytometry following the staining of the cells with anti-glycosphingolipid antibodies [16]. In accordance with previous studies we observed a 4–5 reduction in GM3 levels following treatment of the cells with 10 μM PPMP for 48 h (data not shown). Phospholipid and cholesterol composition of the cells were unchanged following PPMP treatment as shown by our previously studies [17]. Figure 3 shows the cell surface expression levels of CD4, CXCR4 and CCR5 on control and PPMP treated cells. Although there was no significant change in cell surface expression of CD4 or CXCR4, the level of CCR5 expression was reduced by about 50% following treatment of these cells with PPMP consistent with our previous findings [17].

### Effects of PPMP treatment on HIV-1 Env-mediated fusion

**Figure 4:**
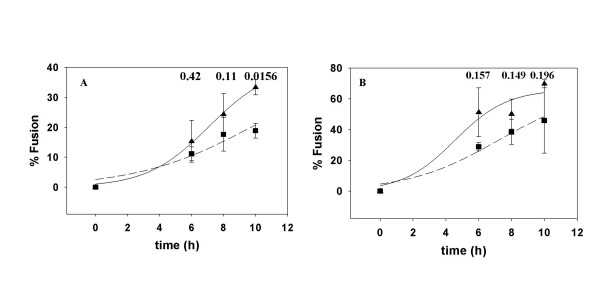
**Effect of PPMP treatment on HIV-1_8x _Env-mediated Fusion**. HIV-1_8x _Env mediated fusion with NIH3T3CXCR4 (A) and NIH3T3CD4 CXCR4 (B) was monitored at different times with untreated (triangles) and PPMP-treated (squares) targets. PPMP treatment and fusion are described in the legends to Figures 3 and 2, respectively. The data were fit to the equation f = a/(1+exp(-(t-t_1/2_)/b)) using Sigmaplot (SPSS, Chicago), where a is the maximal extent, b represents the rate, and t_1/2 _is the time at which the half-maximal fusion extent is reached. The curve fitting yielded the following t_1/2 _values for untreated and PPMP-treated cells, respectively: 7.1 h and 9.8 h (A); 4.6 h and 7.2 h (B). The numbers above the data are p values determined by a paired student t test using Sigmaplot (SPSS, Chicago).

**Figure 5:**
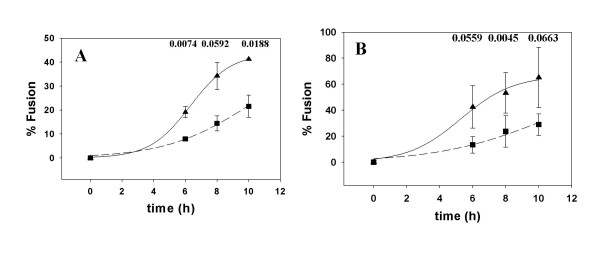
**Effect of PPMP treatment on HIV-1_8xV3BAL _Env-mediated Fusion**. HIV-1_8xV3BAL _Env mediated fusion with NIH3T3CCR5 (A) and NIH3T3CD4CCR5 (B) was monitored at different times with untreated (triangles) and PPMP-treated (squares) targets. PPMP treatment and fusion are described in the legends to Figures 3 and 2, respectively. The data were fit to the equation f = a/(1+exp(-(t-t_1/2_)/b)) using Sigmaplot (SPSS, Chicago), where a is the maximal extent, b represents the rate, and t_1/2 _is the time at which the half-maximal fusion extent is reached. The curve fitting yielded the following t_1/2 _values for untreated and PPMP-treated cells, respectively: 6.3 h and 10 h (A); 5.3 h and 10.5 h (B) The numbers above the data are p values determined by a paired student t test using Sigmaplot (SPSS, Chicago).

Cells expressing the 8x and 8xV3BAL variants of HIV-1_IIIB _were incubated at different times with NIH3T3 cells bearing CXCR4 and CCR5, respectively, with or without CD4. Fusion was monitored before and after treatment of the cells with PPMP. Although the Env constructs we used did not require CD4 for fusion activity it appeared that, in the case of the CXCR4 construct, presence of CD4 on the target cells enhanced the fusion reaction (Figure 4), presumably as a result of better attachment or more robust conformational changes, In the case of the CCR5 construct, presence of CD4 on the target cell did not speed up the fusion reaction significantly (Figure 5). However, there was a significant difference in fusion mediated by the CCR5 construct at the different time points between untreated and PPMP-treated target cells (Figure 5) as indicated by the p values determined by paired student t tests. However, this reduction in fusion activity can be explained by the reduced levels of cell surface CCR5 (Figure 3). PPMP did not have a significant effect on fusion mediated by the CD4-independent CXCR4 construct as indicated by the p values determined by paired student t tests (Figure 4). Collectively, these data indicate that PPMP-treatment does not affect the one-step fusion pathway mediated by HIV-1 Env.

### Effects of cholesterol and PPMP treatment on chemokine receptor function

**Figure 6:**
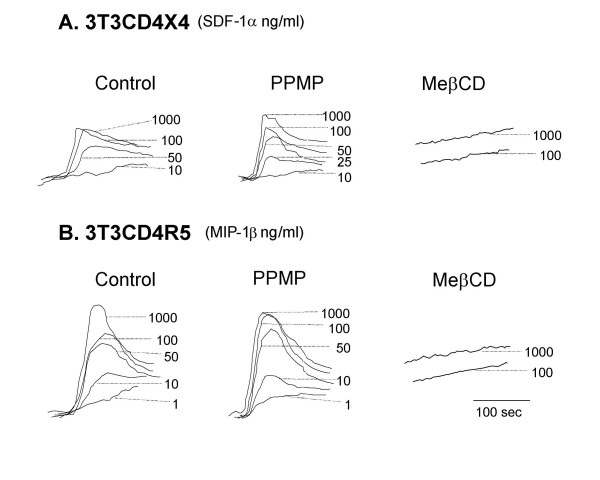
**Chemokine-Triggered Ca^2+ ^Mobilization**. Intracellular Ca^2+ ^mobilization was measured by stimulating Fura-2 loaded NIH3T3CD4CXCR4 (A) or NIH3T3CD4CCR5 (B) cells with chemokines SDF1-α (A) or MIP1-β (B) at different concentrations. The ratio of fluorescence at 340 and 380 nm was calculated using a FL Win Lab program (Perkin-Elmer). Left panel: untreated cells, middle panel: PPMP-treated cells; right panel: MβCD-treated cells.

Nguyen and coworkers showed that removal of cholesterol from the cell membrane results in loss in ligand binding which is likely due to conformational changes in CXCR4 [18] or CCR5 [19]. In accordance with those results, Figure 6 shows that Ca^2+ ^mobilization in NIH3T3 cell lines expressing CXCR4 or CCR5 in response SDF-1α or MIP-1β, respectively, was inhibited following treatment of the cells with MβCD. If treatment of these cell lines with PPMP has a similar effect on ligand binding, we would expect Ca^2+ ^mobilization in NIH3T3 cell lines expressing CXCR4 or CCR5 in response SDF-1α or MIP-1β, respectively, to be inhibited following treatment with PPMP. By contrast, treatment of these cells with PPMP had no effect on chemokine receptor signalling (Figure 6). Thus, the cholesterol effect can be explained by changes in chemokine receptor disposition that leads to engagement with HIV-1 Env.

## Discussion

The two-step model for HIV-1 Env-mediated fusion [1] led to the notion that it is possible to interfere with the two receptor choreography by re-arranging the dance floor. The notion that cholesterol plays a key role in the maintenance of membrane organization [20] has led to a plethora of studies on the role of cholesterol in HIV-1 entry [10,13,18,21-31]. It has been hypothesized that lipid raft domains [12] serve as sites that facilitate receptor interactions and signalling processes, and promote the cooperative event of HIV fusion. However, a role for rafts in HIV-1 entry has been called in question since CD4 mutants that localize this HIV-1 receptor to non-raft domains are perfectly capable of supporting HIV-1 entry [26,27]. In this study we show that removal of cholesterol leads to inhibition of the one-step mode of HIV-1 Env fusion mediated by a CD4-independent strain of HIV-1 (Figure 2). The crucial step is the attachment of HIV-1 gp120 with CXCR4 or CCR5 inducing a barrage of conformational changes in HIV-1 Env, which leads to HIV-1 gp41 six helix bundle formation and fusion. Nguyen and co-workers have shown that maintenance of proper levels of cholesterol in the membrane is essential for chemokine binding and the conformational integrity of CCR5 [19] as well as CXCR4 [18]. Our data on the effects of cholesterol removal on signalling of CCR5 and CXCR4 (Figure 6) are consistent with those results. Therefore, the most straightforward explanation for our data showing inhibition of CD4-independent HIV-1 Env mediated fusion by cholesterol removal is that the conformational integrity of CCR5 and CXCR4 is impaired by this treatment. Therefore, their efficiency in grabbing HIV-1 gp120 and inducing the barrage of conformational changes in HIV-1 Env necessary for fusion has severely been reduced.

The situation with PPMP [15], which inhibits the synthesis of GlcCer, the precursor for glycosphingolipids synthesis, is somewhat more complex. Our initial hypothesis was that glycosphingolipids play a role in HIV-1 entry/fusion. Therefore, a reduction of glycosphingolipid levels by blocking their synthesis would adversely affect HIV-1 fusion. However, glycosphingolipids-deficient cells lines, which were engineered to express HIV-1 receptors, were highly susceptible to HIV-1 Env-mediated fusion [32] suggesting little or no involvement of glycosphingolipids in fusion of cells expressing high levels of HIV-1 receptors. However, PPMP has a number of different effects on sphingolipid metabolism, which include an increase of ceramide levels [33,34]. Zimmerberg and co-workers performed a very detailed study on the effects of PPMP-treatment of cells on the membrane distribution of influenza hemagglutinin [8]. Fluorescence spectroscopy indicated that such treatment alters the relative distance and orientation of these membrane-embedded proteins on molecular scale (6–7 nm), and quantitative electron microscopy indicated relatively small effects on longer (≥ 20 nm) length scales. A similar study on CD4 and co-receptor distribution is beyond the scope of this study. However, CD4 has a similar domain structure as hemagglutinin (large extracytoplasmic domain, one membrane-spanning region, short cytoplasmic tail) and is also considered to be a "raft" protein [35]. We may therefore surmise that PPMP treatment will exert a similar effect on CD4 distribution on the cell surface. On the other hand, the seven trans-membrane proteins CXCR4 and CCR5 may be less sensitive to such treatment as indicated by the Ca^2+ ^signaling experiment (Figure 6). Therefore, the sensitivity to PPMP treatment in the CD4-independent setting may be less pronounced when a one-step mechanism is required to mediate HIV-1 Env fusion.

## Conclusion

By reducing HIV-1 Env-mediated fusion to a one-step chemokine receptor- dependent process, we have shown that cholesterol plays a role in both CD4 and chemokine receptor-mediated steps, whereas modulation of sphingolipids only affects the choreography between the two steps. Chemokine receptor function remained intact following treatment of cells with PPMP. Therefore such treatment may be considered a more suitable agent to inhibit CD4 dependent HIV-1 infection.

## Methods

### Materials

Phycoerythrin (PE)-labeled Mabs against CD4, CXCR4 and CCR5 and their isotype controls were obtained from Pharmingen (San Diego, CA). The anti-GM3 Mab GMR6 was obtained from Seikagaku America (Falmouth, MA) and Cy3-conjugated anti-mouse Fab from Jackson Immunochemicals (West Grove, PA). Methyl-β-cyclodextrin (MβCD) and cholesterol/MβCD complexes purchased from Sigma (St. Louis, MI), and 1-phenyl-2-hexadecanoylamino-3-morpholino-1-propanol (PPMP) from Matreya, Inc. (Pleasant Gap, PA). Cholesterol in total cell lysate was determined using the Cholesterol Oxidase kit (Wako Chemicals USA Inc, Richmond, VA). The cytoplasmic dyes 5-chloromethylfluorescein diacetate (CMFDA), 5- and 6-([(4-chloromethyl)benzoyl]-amino)tetramethyl-rhodamine (CMTMR) and Fura-2 AM were from Molecular Probes (Eugene, OR). The chemokine receptor ligands SDF1-α and MIP1-β were from Peprotech (Rocky Hill, NJ). All other biochemicals used were of the highest purity available and were obtained from regular commercial sources.

### Cells and viruses

NIH 3T3 cells constitutively expressing CD4 and/or CXCR4, and NIH 3T3 cells constitutively expressing CD4 and/or CCR5, were kindly provided by Dr. Dan Littman (New York University, New York). HIV-1 Env 8x and 8xV3BAL [36], kindly provided by Dr. Robert Doms, University of Pennsylvania, Philadelphia, were expressed on the surface of NIH3T3 cells by transfection using the vaccinia T7-polymerase (vTF7-3), which was obtained through the AIDS Research and Reference Reagent Program, Division of AIDS, NIAID, NIH from Drs Tom Fuerst and Bernard Moss.

### Surface expression of GM3, CD4 and chemokine receptors

The fluorescence of cells stained with PE-conjugated Mabs against CD4, CXCR4 and CCR5 was compared with the appropriate PE-conjugated isotype controls. Cell labeling with PE-conjugated Mabs was done in the presence of 2% serum, after which cells were washed and fixed with 1% paraformaldehyde. The analysis was performed on a FACStar plus flow cytometer. GM3 levels were monitored by immunofluorescence using the anti-GM3 Mab (GMR6) and a Cy5-stained antimouse Fab.

### HIV-1 Env-mediated cell fusion

To express 8x or 8x-V3BaL gp120-gp41, NIH3T3 cells plated on T75 flasks were incubated with the vaccinia virus vTF7-3 (5 M.O.I.) for 1–2 hrs at 37°C. Subsequently, the cells were transfected with 15 μg DNA (psp73.8x or psp73.8x.V3BaL using lipofectamine (Invitrogen, Inc.) in 3 ml DMEM (without serum) and incubations were continued for 4–6 hrs at 37°C. DMEM containing 10% FBS + antibiotics (D10) was added and cells were incubated for additional 16–20 hours. For the fusion assay, cells were harvested using EDTA-based cell dissociation buffer (Life Technologies, Inc.) and labeled with 10 μM CMFDA following manufacturer's directions. The CMFDA-labeled HIV-1 Env-expressing cells (2 × 10^5 ^per well) were added to the same number of CMTMR-labeled NIH3T3 targets expressing CD4 and/or cognate co-receptors and the samples were incubated for 4–10 hrs at 37°C. Dye redistribution as a result of fusion was monitored microscopically as described previously [37]. The extent of fusion was calculated as: Percent Fusion = 100 × number of cells positive for both dyes/number of bound cells positive for CMTMR.

### Calcium mobilization

Intracellular [Ca^2+^] mobilization was measured by incubating 2 × 10^7 ^cells/ml in loading medium (DMEM containing 10% FCS and 2 mM glutamine) with 7 mM Fura-2 AM for 45 min at room temperature. The dye-loaded cells were washed and resuspended in saline buffer (138 mM NaCl, 6 mM KCl, 1 mM CaCl2, 10 mM HEPES, 5 mM glucose, and 0.1% BSA, pH 7.4) at a density of 0.5 × 10^6^/ml. The cells were then transferred into quartz cuvettes (1 × 10^6 ^cells in 2 ml saline buffer), which were placed in a fluorescence spectrometer (Perkin-Elmer, Beaconsfield, U. K.). Chemokines were added in a volume of 20 ml to each cuvette. The intensity of the fluorescence was measured as the ratio at 340 and 380 nm wavelengths and calculated using a FL WinLab program (Perkin-Elmer).

## Competing interests

The author(s) declare that they have no competing interests.

## Authors' contributions

SA, SSR, MV and AP performed experiments on cholesterol levels, HIV-1 receptor expression and HIV env-mediated fusion. JMW performed the Ca^2+ ^signaling experiments. RB conceived of the study, participated in its design and coordination and wrote the manuscript. All authors read and approved the final manuscript.
